# 321. Early Intervention: Readmission Reduction Efforts in OPAT via Early Patient Contact

**DOI:** 10.1093/ofid/ofad500.392

**Published:** 2023-11-27

**Authors:** Michael Swartwood, Asher J Schranz, Claire E Farel, Nikolaos Mavrogiorgos, Julia B Fabricio, M C Bowman, Teresa M Oosterwyk, Renae Boerneke

**Affiliations:** University of North Carolina Medical Center, Chapel Hill, North Carolina; University of North Carolina, Chapel Hill, NC; UNC Chapel Hill, Chapel Hill, North Carolina; University of North Carolina Medical Center, Chapel Hill, North Carolina; University of North Carolina Medical Center, Chapel Hill, North Carolina; UNC Medical Center, Chapel Hill, North Carolina; UNC Medical Center, Chapel Hill, North Carolina; UNC Health, Chapel Hill, North Carolina

## Abstract

**Background:**

Outpatient parenteral antimicrobial therapy (OPAT) leverages the expertise of a multidisciplinary Infectious Diseases (ID) team to provide antimicrobial therapy at home in a safe and cost-effective manner. To reduce adverse events (AE) and hospitalizations among patients receiving OPAT, we implemented two initiatives: early care coordination phone calls (CCPC) and short-term ID follow-up appointments after OPAT initiation. The difficulties with hospital discharge are well described. Our efforts were designed to prevent these before they resulted in a hospital readmission.

**Methods:**

In March 2019, we implemented an early CCPC after hospital discharge. The content of these conversations were chosen to identify or reduce potential reasons for readmission. Patients at potentially increased readmission risk were prioritized via specific criteria.

In October 2020, we aimed to shorten the interval between hospital discharge and ID follow-up. Historically, these had taken place at the end of OPAT therapy. By shortening this interval, we hoped to leverage ID expertise to identify potential rehospitalization during OPAT.

**Figure:**
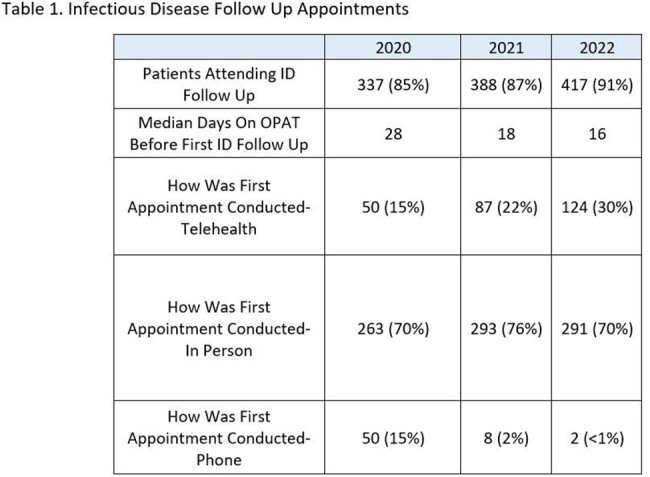

**Figure:**
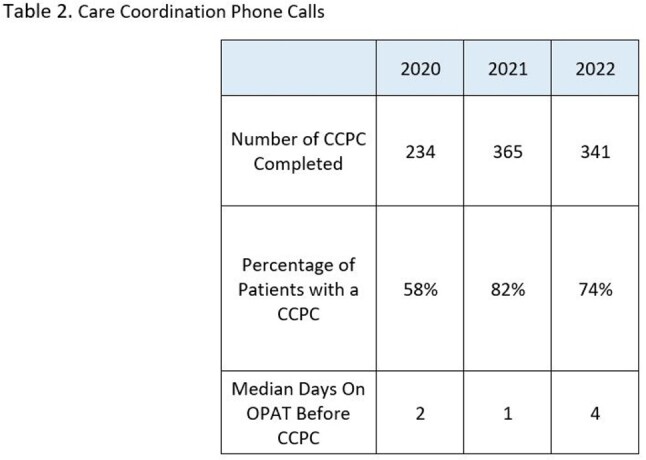

**Results:**

For patients completing OPAT therapy in 2021, 82% received a CCPC. While the call content was clinically beneficial in many cases, the readmission rate was static.

For 2020, the median days until ID follow-up was 28 days and decreased to 16 days in 2022. The percentage of visits via telehealth increased from 15% in 2020 to 30% in 2022.

During 2015-2019, the readmission rate during OPAT was 19%. In 2022, UNC OPAT achieved a readmission rate of 14%. This was a 9% readmission rate for reasons unrelated to OPAT and a 5% readmission rate for OPAT-related causes.

The reduction in negative patient outcomes was not limited to readmissions. For patients completing OPAT in 2022, we saw a decrease in the rate of patients experiencing several AE’s.

**Figure:**
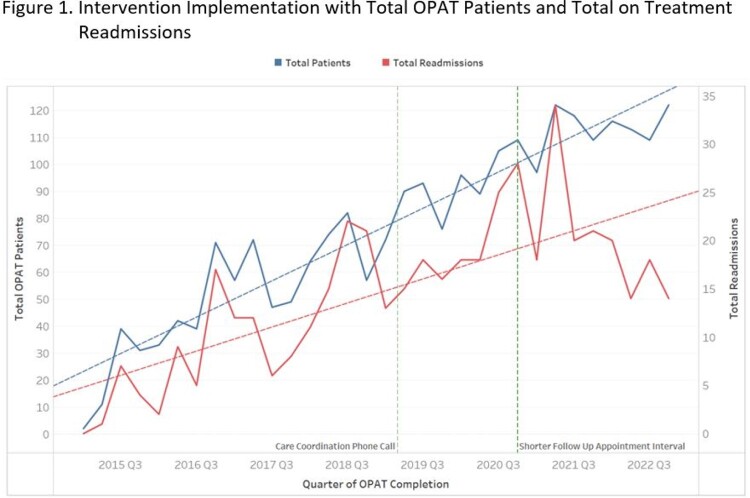

**Figure:**
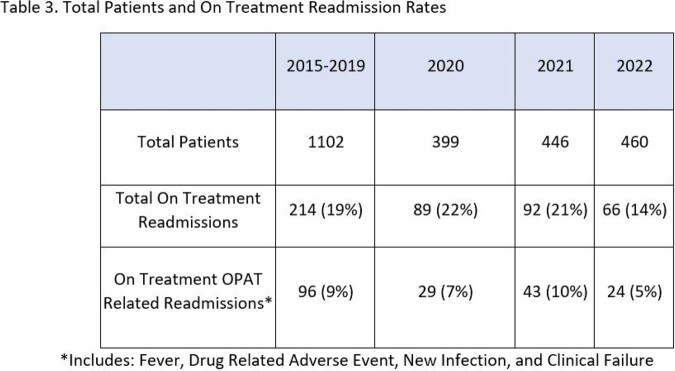

**Figure:**
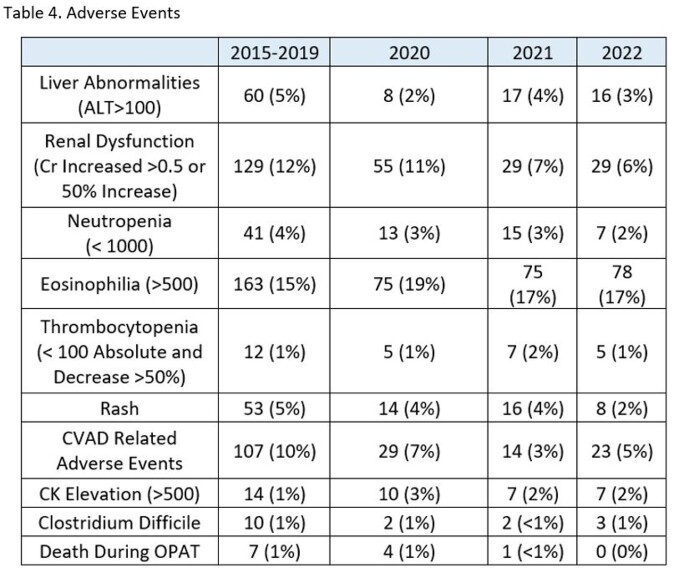

**Conclusion:**

We piloted two interventions to decrease hospitalizations during OPAT. While early CCPC had qualitative benefits, it did not correlate with a decrease in hospitalizations. Early ID appointments did correlate with a large reduction in on-treatment readmissions. These findings are consistent with numerous studies showing that early ID clinician involvement is linked to favorable outcomes for patients and health systems.

**Disclosures:**

**Asher J. Schranz, MD, MPH**, WoltersKluwer: Honoraria

